# Mental Health and Well-Being Among Individuals With a Sensory Loss During COVID-19 Lockdown Measures

**DOI:** 10.1093/geroni/igab046.1580

**Published:** 2021-12-17

**Authors:** Natascha Merten, Amy Schultz, Matthew Walsh, Suzanne van Landingham, Paul Peppard, Carol Ryff, Kristen Malecki

**Affiliations:** 1 University of Wisconsin - Madison, Madison, Wisconsin, United States; 2 University of Wisconsin-Madison, Madison, Wisconsin, United States

## Abstract

Hearing and vision impairment are highly prevalent chronic conditions and are associated with poorer mental health and well-being. Mental health problems may be exacerbated by COVID-19-related lockdown measures and limitations of in-person contacts may affect those with sensory impairments more severely. We aimed to determine whether hearing and/or visual impairment were associated with worse mental health and psychological well-being during lockdown measures in Spring/Summer 2020 in Wisconsin. We included 1341 (64% women, aged 20-92 years) Survey of the Health of Wisconsin participants of a COVID-19 survey (May-June, 2020). We assessed self-reported current mental health and psychological well-being and vision and hearing impairment. Logistic regression models with vision and hearing impairments as determinants and multiple mental health and well-being outcomes were used and adjusted for age, gender, race, education, heart disease, hypertension, hyperlipidemia and diabetes. In preliminary analyses, we found associations of vision impairment with increased odds of generalized anxiety disorder (odds ratio=2.10; 95% confidence interval=1.32-3.29) and depression (2.57; 1.58-4.11). Individuals with a vision impairment were more likely to be taking medication for depression (1.75; 1.13-2.68), report being lonely (1.65; 1.00-2.64) and report hopelessness (1.45; 1.01-2.08). Individuals with a hearing impairment were more likely to be taking depression medications (1.72; 1.07-2.73) and to report being lonely (1.80; 1.05-2.98). Sensory impairment was not associated with stress levels or sense of purpose in life. Individuals with sensory impairment may represent a particularly vulnerable population during the COVID-19 pandemic. Future research should determine underlying reasons and interventions to mitigate this populations’ disadvantages.

